# Knowledge and Attitude Towards Cardiopulmonary Resuscitation Among Doctors of a Tertiary Care Hospital in Karachi

**DOI:** 10.7759/cureus.4182

**Published:** 2019-03-06

**Authors:** Aamina Majid, Momal Jamali, Muhammad Moinuddin Ashrafi, Zaiyn Ul Haq, Rafia Irfan, Aiman Rehan, Muhammad Mustafa Memon, Muhammad A Khan, Jai Kumar, Pankaj Kumar Singh, Sushil Allen Luis, Pradhum Ram, Savita Lasrado, Fouzia Imtiaz, Ritesh G Menezes

**Affiliations:** 1 Internal Medicine, Dow University of Health Sciences, Karachi, PAK; 2 Forensic Medicine, Kathmandu University School of Medical Sciences, Kathmandu, NPL; 3 Cardiology, Mayo Clinic, Rochester, USA; 4 Internal Medicine, Albert Einstein Medical Center, Philadelphia, USA; 5 Internal Medicine, Father Muller Medical College, Mangalore, IND; 6 Genetics, Dow University of Health Sciences, Karachi, PAK; 7 Pathology, Imam Abdulrahman Bin Faisal University, Dammam, SAU

**Keywords:** resuscitation, coronary artery disease, acute coronary syndrome, public health

## Abstract

Objective

Cardiac arrest is an emergency, which can be managed effectively by sound knowledge and practice of basic life support (BLS) skills. However, it has been globally reported that the knowledge of doctors regarding cardiopulmonary resuscitation (CPR) and BLS is sub-standard. We conducted this study with the aim to assess the knowledge and attitude of doctors toward CPR in Dr. Ruth K.M. Pfau Civil Hospital, one of the largest tertiary care hospitals, in Pakistan.

Methods

We conducted a cross-sectional study, in Dr. Ruth K.M. Pfau Civil Hospital located in Karachi, Pakistan, using cluster sampling. A total of 285 doctors were interviewed.

Results

A majority of the doctors were unaware of the revised rate and depth of chest compressions (65.6% and 75.8% respectively). While many know the abbreviations of BLS and CPR (96.55% and 95.4%, respectively), 56.5% did not know what automated external defibrillator (AED) stood for. CPR was preferred over chest compression-only resuscitation (CCR) by 91.6% of the doctors. Half of the participants rated their knowledge as average. Most stated that they will not be reluctant to perform CPR in an emergency situation. The majority also agreed that BLS training should be an integral part of the medical curriculum.

Conclusion

There is an evident lack of knowledge of CPR among healthcare professionals, particularly regarding the updates made in the 2015 American Heart Association (AHA) guidelines. However, an overall positive attitude was observed.

## Introduction

Cardiac arrest remains a huge medical problem as well as a public health concern. Despite all the recent advances, it is a leading cause of death in most parts of the world. In the United States alone, annually 300,000 cases of out of hospital cardiac arrest (OHCA) are reported, while in Europe around 350,000 individuals die following OHCA [1-2].

Cardiac arrest can occur both inside and outside the hospital setting, which necessitates the need for early recognition and treatment. It is possible to reduce the high mortality rate associated with cardiac emergencies by ensuring adequate knowledge and practice of basic life support (BLS) skills. The American Heart Association (AHA) has issued comprehensive guidelines for both in and out of hospital management, adult cardiac arrest chain of survival, immediate recognition of cardiac arrest, early activation of emergency medical services (EMS), early cardiopulmonary resuscitation (CPR), and defibrillation [3].

CPR, in particular, is a simple maneuver which if performed correctly can greatly increase the likelihood of return of spontaneous circulation (ROSC) and survival. As healthcare professionals encounter several life-threatening emergencies on a daily basis, they are expected to have a profound knowledge of the CPR guidelines. However, many junior doctors are not capable of performing CPR effectively [4]. The inadequate knowledge of resuscitation has been reported globally. Studies from India, Turkey, Greece, Nigeria, and Nepal also cite a lack of knowledge regarding CPR among healthcare professionals [5-12]. In addition, the absence of BLS and CPR inclusion in the medical school curriculum is also an important contributing factor [13-14].

Data from Pakistan also suggests that awareness of BLS and CPR among students, doctors, and nurses is very poor [10,13,15]. Lack of knowledge and information about CPR can seriously affect clinical outcomes and lead to medico-legal complications. On the other hand, inappropriate technique and poor knowledge can become counterproductive as they are liable to cause CPR-related injuries [16].

Multiple studies have been conducted regarding public awareness of CPR but there are only a handful of studies evaluating the knowledge among health care professionals. Even fewer studies have included evaluation of the updated guidelines and covering the aspect of a doctor’s attitude about CPR. Considering the scarcity of data in Pakistan, we carried out a survey in Dr. Ruth K.M. Pfau Civil Hospital, Karachi with the primary objective of assessing the level of awareness of CPR among healthcare professionals and their attitude towards it.

## Materials and methods

Sample size and study design

The sample size was calculated from Open Epi using an anticipated frequency of 50%. It was found to be 285. Cluster sampling was selected as the study design. All the different wards in the hospital were designated as clusters, and a fixed number of doctors were randomly selected from each cluster.

Inclusion and exclusion criteria

The doctors on morning duty in their respective wards during the time of data collection were included in the study. The study sample included house officers, post-graduate residents, assistant professors, professors, and heads of departments. Doctors posted in the emergency department, out-patient departments, and operation theaters were excluded from this study in order to prevent any inconvenience to doctors or patients.

Study tool

We used a structured questionnaire consisting of 24 questions as the study tool. The questionnaire had been designed by the study team and tested in a pilot study. No question that would reveal the identity of the person was incorporated. Confidentiality regarding personal information was maintained throughout. The questionnaire was divided into three sections: demographics, knowledge, and attitude.

a) The demographics included the background characteristics of the doctors including their age, designation, years of clinical experience, and the number of times they had witnessed or performed CPR.

b) In the knowledge section, respondents were asked to answer eight questions regarding CPR. Questions were based on the AHA 2015 guidelines, with a maximum score of eight.

c) In the last section, the attitude of the participants toward CPR was gauged including their confidence in performing CPR and hesitance toward mouth-to-mouth ventilation. Their opinion regarding the inclusion of BLS in the academic curriculum was also sought. Finally, the respondents were asked which method of resuscitation they preferred, chest compression-only resuscitation (CCR) or standard CPR.

Data collection

Data was collected from the study participants by some of the authors in person as an interview. This was conducted from September 2017 to November 2017. Prior written informed consent was obtained. Data that would reveal the identity of the participants in any manner was removed from the study. Additional instructions were provided for certain multiple choice questions to prevent ambiguity.

Data analysis

Data were analyzed using statistical package for social science (SPSS) version 23 for Windows. Simple frequencies and proportions were calculated. Because the data were not normally distributed, the Mann-Whitney test and Kruskal Wallis test were used for the comparison of two or more groups. Knowledge was assessed based on scores obtained (out of a maximum, eight) in questions pertaining to CPR specifically, while attitudes were interpreted using 4-5 point Likert scales.

## Results

A total of 285 doctors were approached for the study. All of them consented to be a part of this study. The demographic characteristics of the participants are shown in Table 1. Although a large proportion had witnessed CPR being performed (*n *= 260, 91.2%), a quarter had never given CPR before (*n *= 71, 24.9%).

**Table 1:** Study participants characteristics RMO, resident medical officer; BLS, basic life support; AHA, American Heart Association

Characteristics	Frequency	Percent (%)
Age Group		
20-29 years	253	88.8
30-39 years	27	9.4
40-49 years	2	0.70
50 years and above	3	1.0
Gender		
Male	104	36.5
Female	181	63.5
Designation		
House officer	139	48.8
RMO	4	1.4
Post Graduate	135	47.4
Assistant Professor	5	1.7
Professor	2	0.7
Experience (in years)		
<2	161	56.5
3-5	101	35.4
6-10	16	5.6
>11	7	2.5
Attended a BLS course		
Yes	239	83.9
No	46	16.1
Awareness of the AHA 2015 guidelines		
Yes	114	40
No	171	60
Total	285	100

The responses to the questions are shown in Table 2. Surprisingly, the rate and depth of chest compressions revised in the 2015 AHA guidelines were incorrectly answered by majority of the doctors. Two-thirds were not aware of the revised rate of chest compressions (*n *= 187, 65.6%). Similarly, only a quarter of the study group knew the correct depth of chest compressions (*n *= 69, 24.2%). This information is essential for delivering high-quality CPR. Alarmingly, 60% had not updated themselves with the 2015 AHA guidelines (*n *= 171).

**Table 2:** CPR knowledge of the participants BLS: basic life support; CPR: cardiopulmonary resuscitation; AED: automated external defibrillation

Questions	Correct (%)	Incorrect (%)
1. BLS stands for	275 (96.5)	10 (3.5)
2. CPR stands for	272 (95.4)	13 (4.6)
3. AED stands for	124 (43.5)	161 (56.5)
4. CPR can be performed in hospitals only or both in and out of hospital?	259 (90.9)	26 (9.1)
5. Location of chest compressions in adults	191 (67)	94 (33)
6. Rate of chest compressions per minute during adult CPR	98 (34.4)	187 (65.6)
7. Ratio of chest compressions to ventilation in adults	233 (81.7)	52 (18.2)
8. Depth of chest compressions in adults during CPR	69 (24.2)	216 (75.8)

The percentage of the correct answers varied from 25.2% to 96.5%. The mean score for the participants was 5.35 with a median score of 5. Of the 285 participants, the majority scored more than four. However, only 3.5% achieved a maximum score of eight. The highest mean score (6.0 ± 1.41) was obtained by the RMOs, who spent much of their time inward.

An overwhelming majority (91.6 %) preferred the standard CPR to CCR. The reason given for the above preference was the effectiveness of CPR (45.6%), recommended by the National Resuscitation Council (42.9%) and confidence in the CPR techniques (9.6 %). Almost all (*n *= 279, 97.7%) recommended the inclusion of BLS in the academic curriculum. Half of the responders (*n *= 127, 44.6%) thought that the general public should be trained for CPR. When asked to grade themselves on their knowledge on BLS, around half of the participants (*n *= 149, 52.3%) felt it was average, while a third (*n *= 95, 33.3%) said it was good. In case of an emergency, a majority (*n *= 261, 91.6%) would not be hesitant to perform CPR, but only 17.9% were willing to give mouth to mouth ventilation without any barrier. Two-thirds (*n *= 195, 68.4%) preferred to use some type of barrier, while 6.3% (*n *= 18) refused to do it at all.

The scores were compared according to the different characteristics of the participants, as shown in Table 3. There was no association between the knowledge scores of the participants with their age or years of experience. It was also found out that the mean knowledge scores were not statistically different in the participants of various designations. Surprisingly, the professors were found to have the least mean knowledge score.

**Table 3:** Comparison of the scores in relation to the different characteristics of the participants RMO: resident medical officer

Demographics	Number	Mean score	Standard deviation	P value
Age (years)				
20-29	253	5.36	1.29	0.405
30-39	27	5.33	0.58	
40-49	2	5.00	0.00	
50 and above	3	4.00	1.41	
Duration of clinical work (years)				
<2	161	5.27	1.48	0.462
3-5	101	5.41	1.23	
6-10	16	5.81	0.91	
>11	7	5.17	1.72	
Designation				
House officer	139	5.46	1.42	0.052
RMO	4	6.00	1.41	
Post Graduate	135	5.25	1.33	
Assistant Professor	5	4.75	0.96	
Professor	2	4.00	1.41	

## Discussion

CPR is not only a component and backbone of BLS, but it is also a commonly performed lifesaving procedure in a commonly encountered emergency situation. While CPR can itself save a life, its timely, effective, and high-quality administration is extremely important in relation to the outcome. Healthcare professionals including emergency medical technicians, paramedics, and even qualified by-standers are expected to respond and deliver CPR. However, problems with the retention of knowledge and skills and outdated information need to be identified and addressed. It is also safe to say that a doctor’s perspective and ethical views can also dictate their CPR administration.

Our study revealed that although the doctors are expected to manage cardiopulmonary arrest, they have gaps in their knowledge. Although the majority of the participants scored more than four out of a maximum of eight, there were still 22.8%, who scored less than four. Considering these were basic and fundamental questions regarding CPR, it is a serious concern that the doctors missed any one of them. Only 3.5% achieved a perfect score, a very low figure. This confirms the results of earlier studies among different healthcare professionals, even among cardiologists alone [5,7-8,13-14,17]. This scarcity of knowledge is perhaps due to the lack of structured training of BLS/CPR in medical schools. Also, the busy residency schedules and the lack of resources might make it extremely difficult for doctors to learn the skills of resuscitation in clinical settings. Incorrect answers from the senior doctors, who have long completed their medical school, show that they lagged behind in their awareness of BLS as well. The significance of refresher courses here cannot be stressed enough and is the need of the hour. Benden et al. and Goodwin identified that refresher training should take place every six months to maintain proficiency in CPR [18-19].

It is known that CPR together with early delivery of shock with a defibrillator can improve survival rates to as high as 75% [20]. However, in our study, only 43.5% knew what AED stood for. This is similar to another study in which only 33% were aware of the abbreviation [8]. This could be due to the lack of availability of AED in hospitals, making it less likely for the doctors to come across one in their daily practice.

The rate and depth of chest compressions, revised by AHA in 2015, was incorrectly answered by a majority of the respondents. Nearly two-thirds of our study populations (65.6%) failed to identify the correct rate, which was similar to that of a study in India by Chaudhari et al. (64.31%) [7]. This is accounted by the fact that more than half of the doctors had not updated themselves to the new guidelines, which also parallels the figure in the aforementioned study. In another study, only 1.2%, 2.2%, and 13% of the participants knew the number, rate, and depth of compressions, respectively [21].

The low overall score achieved by the professors coincides with the study by Zamir et al., where the senior doctors showed decreased knowledge as compared to the junior doctors [15].

Attitude plays an essential role especially when it comes to starting the CPR process. In our study, the participants had a generally positive attitude toward CPR. They were eager to perform CPR in emergency situations despite having knowledge gaps. This is consistent with the results of a study that has shown that despite the lack of knowledge, participants were willing to perform CPR [10]. The majority recommended the inclusion of BLS courses in the curriculum. CCR has been introduced as an alternative to standard CPR for bystanders. It is argued that CCR overcomes reluctance to perform mouth-to-mouth ventilation and is easier to teach [22]. In our study, a majority preferred the standard CPR over CCR and most reasoned that it was more effective. This contrasts with Ong et al.’s study where majority of the general practitioners preferred CCR to CPR [23]. Although the majority claimed that they would not feel hesitant to administer CPR, they preferred to use some type of barrier when giving ventilation. This might be due to some religious restraints of the participants. It can also be explained by the fear of respondents of acquiring airborne and other infections. Our results also illustrate that most participants felt that their knowledge regarding CPR was average. Although a study showed no correlation between knowledge scores and participants self-appraisal, it is argued that a doctor’s lack of confidence would negatively affect to lead resuscitation [5]. Approximately 83.9% of the respondents had attended a BLS course that is higher than the figures reported in other studies [24-25].

It is essential to standardize training in BLS as well as adult life support (ALS) and make it a mandatory component of all medical, nursing, and paramedical school undergraduate curriculum. Zamir et al. showed that attending a CPR class has a positive effect on theoretical knowledge [15]. The teachers, school children, public, and all lay persons from a community should also be trained for BLS and first aid so that they can respond to a crisis correctly. Short refresher courses can be arranged for those who already have taken a course previously to spare the funding and assure revision. Chaudhary et al. demonstrated how hands-on training improved the knowledge and techniques by comparing pre-test assessment and post-workshop scores [26]. BLS training at regular intervals having hands-on practice and practical demonstration should be implemented. This should involve all the health care professionals irrespective of their qualification, experience, and specialty. The doctors should be regularly updated for new guidelines. Furthermore, measures should be taken to involve the general public, in order to increase the number of first aid responders.

## Conclusions

The knowledge regarding CPR was found to be incomplete and inadequate among the doctors. However, a positive attitude was noted in our study. Further CPR training, including initial training and subsequent refresher courses, should be considered to address deficiencies.
